# Comparison of Outcomes Between Radical Radiotherapy and Radical Cystectomy in Muscle Invasive Bladder Cancer in a Cancer Specialized Unit of a Developing Country

**DOI:** 10.7759/cureus.10057

**Published:** 2020-08-26

**Authors:** Shaukat Fiaz, Azfar Ali, Siddique Adnan, Muhammad Arshad Irshad Khalil, Yasir Rasheed, Muhammad Abu Bakar, Zubair Ahmad Cheema, Khurram Mir

**Affiliations:** 1 Surgical Oncology, Shaukat Khanum Memorial Cancer Hospital and Research Center, Lahore, PAK; 2 Urology, Instutute of Kidney Diseases Hayat Abad Medical Complex, Peshawar, PAK; 3 Urology, Agha Khan Hospital, Karachi, PAK; 4 Cancer Epidemiology and Biostatistics, Shaukat Khanum Memorial Cancer Hospital and Research Center, Lahore, PAK; 5 Urology, Shaukat Khanum Memorial Cancer Hospital and Research Center, Lahore, PAK; 6 Surgical Oncology, Shaukat Khanum Memorial Cancer Hospital and Research Centre, Lahore, PAK

**Keywords:** radical cystectomy, radical radiotherapy, muscle invasive bladder cancer

## Abstract

Introduction

Radical cystectomy (RC) is the current standard of care for treating muscle-invasive bladder cancer (MIBC), however bladder preservation by offering radical radiotherapy (RT) is gaining interest for improving the quality of life while maintaining a reasonable oncological outcome. In this study, we have compared outcomes of the two treatment options.

Materials and methods

This is a 10-year retrospective cohort study that included all patients who were treated for histologically proven muscle-invasive bladder cancer in the department of uro-oncology at Shaukat Khanum Memorial Cancer Hospital and Research Centre from January 2005 to January 2015. Data was analysed using Statistical Product and Service Solutions (SPSS), version 21 (IBM Corp., Armonk, NY). The primary end point of our study was to calculate the three- and five-year disease-free survival (DFS) and overall survival (OS).

Results

A total of 230 patients were included in the study with male gender predominating (88%). The mean and standard deviation for age was 58.32+11.128. Radical cystectomy was performed in 119 patients while 111 received RT. Clinically, 34% had stage 2 disease, while 66 % had stage 3 cancer. The median follow-up duration was 41 months (range: 2-155). During follow-up 57.4% of patients showed no recurrence. Local recurrence was found in 9.6% patients and distant metastasis in 32.2%. The three-year DFS of RC was 63% and that of RT was 57% while the five-year DFS for RC and RT were 60% and 49%, respectively (p=0.196). The three-year OS of RC was 64% and that for RT was 58%. On further analysis the five-year OS of RC was 53% and that for RT was 50% (p=0.98). Upon stage-based comparisons, we found no statistically significant difference between the three- and five-year DFS and OS of stage 2 and stage 3 when treated with either modality.

Conclusion

Most studies favor RC and consider it as the gold standard treatment for muscle-invasive bladder tumor treatment. The current study reveals that bladder preservation approach by chemo radiotherapy is a viable treatment option, having comparable oncological outcomes with patients receiving radical cystectomy, and can be offered to patients having muscle-invasive urothelial bladder cancer.

## Introduction

Globally, bladder cancer is the 11th most common cancer [1]. Muscle invasive urothelial carcinoma has aggressive behavior and prognosis is poor, and if not treated it has a two-year survival lesser than 15% [2]. Although for muscle-invasive bladder cancer (MIBC) radical cystectomy (RC) is considered the standard treatment of choice, bladder preservation modalities have renewed interest especially due to quality of life and morbidity concerns associated with radical cystectomy [3]. Currently, RC with ileal conduit urinary diversion is the most common procedure to treat MIBC [4]. Tri-modality therapy with maximum resection of bladder tumor (TURBT) followed by chemo- and radiotherapy is used as an alternative to preserve the bladder [5]. Regarding oncological outcomes, chemo-RT has shown modest results. Efstathiou et al. [6] noted a complete response (CR) rate of 72%, with a 10-year disease-free survival (DFS) of 59% and overall survival (OS) of 35%, while RC had a 10-year DFS of 66.8% and OS of 44.3% [7]. Another study documented the morbidity associated with chemo-RT. Grade 3-4 toxicities, mainly metabolic, hematological, and genitourinary, related to chemotherapy were 19-35%, while complications like small capacity bladder and/or hematuria associated with radiotherapy were 6-11% [8]. On the other hand, RC can be associated with significant post-surgical complications, as noted by Lowrance and colleagues, who noted them in 41% of patients [9].

The potential for comparable efficacy in survival, coupled with the benefit of bladder preservation, leading to reduced morbidity have driven interest in offering chemo-RT to patients with MIBC. Published literature comparing radical cystectomy with radical radiotherapy for muscle-invasive bladder cancer is scarce. We share our experience and present comparative analysis of these two treatment modalities at our institution in patients with MIBC having stage 2 (T2a/bN0M0) and stage 3 (T3a-4aN0M0) disease.

## Materials and methods

After approval from the Institutional Review Board (IRB number Ex-17-06-19-01), records of all patients aged 18 or older with non-metastatic muscle-invasive bladder cancer (clinical stage 2 cT2a/b N0M0 and clinical stage cT3a-T4a NoM0), who underwent RC and chemo-RT in uro-oncology departments of Shaukat Khanum Memorial Cancer Hospital and Research Center, Pakistan from January 2005 to January 2015 were analyzed retrospectively. Patients with irresectable disease (i.e. cT4b) were excluded.

We have analyzed patient demographics, gender, medical comorbidities, age at initial presentation, modality of treatment, clinical stage of disease, and histopathology.

Patients in the radical cystectomy group were followed with imaging (CT scan or MRI) on regular intervals for any local or distant recurrence. Patients in the radiotherapy group after completion of radiotherapy were followed with both cystoscopy (for local recurrence) and imaging (CT scan/MRI) for metastasis at regular intervals.

Statistical analysis was performed by using IBM Statistical Product and Service Solutions (SPSS) for Windows, version 21 (IBM Corp., Armonk, NY). Continuous variables were stated as mean±standard deviation, and categorical variables were calculated as percentages and frequencies. Estimation of survival was carried out by the Kaplan-Meier method.

## Results

Of the 230 treated patients, 88% were male. The mean and standard deviation of age was 58.32+11.128 as shown in Table 1. Table 2 explains the timing of chemotherapy with modality of treatment. Radical cystectomy was performed in 119 patients and radical radiotherapy in 111. The majority of patients (66%) had clinical stage 3 whereas 34% had clinical stage 2. The median follow-up duration was 41 months (range: 2-155). The number of dropout patients was 15, which did not have a major impact on the results of our study. No recurrence was noted in 57.4%, local recurrence was found in 9.6%, and distant metastasis in 32.2% (Table 3).

**Table 1:** Demographic characteristics of the patients

Table 1: Demographic characteristics of the patients.		
Modality of Treatment	Categories	N (%)
Age (years)	Mean ± standard deviation	58 ± 11
Sex	Male	202 (87.8)
	Female	28 (12.2)
Province	Punjab	101 (43.91)
	Sindh	16 (6.96)
	KPK	81 (35.22)
	Balochistan	20 (8.69)
	Kashmir	12 (5.22)

**Table 2:** Timing of chemotherapy with modality of treatment

Chemotherapy	Radical radiotherapy (n=111)	Radical cystectomy (n=119)
No	08	63
Concurrent	81	_
Neo Adjuvant	22	46
Adjuvant	--	10

**Table 3:** Results of radiotherapy and radical cystectomy

	No Recurrence (N%)	Local Recurrence (N%)	Metastasis (N%)
Radial cystectomy (n=119)	74 (62%)	6 (5%)	39 (33%)
Radical radiotherapy (n=111)	59 (53%)	16 (14%)	36(32%)
	133 (57.4%)	22 (9.6%)	75 (32.2%)

**Figure 1:**
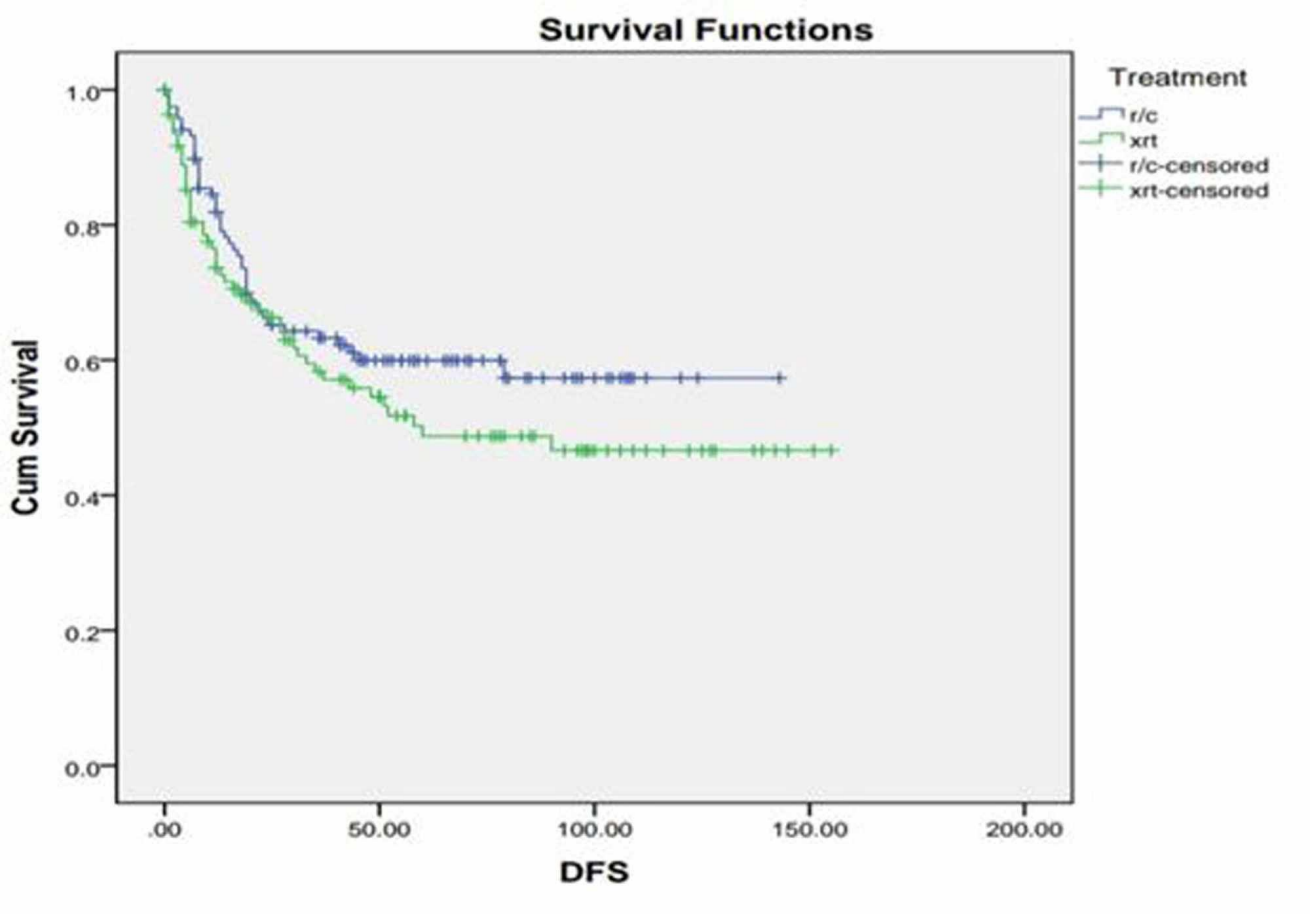
Radical radiotherapy (xrt) vs. radical cystectomy (r/c) disease-free survival

**Figure 2:**
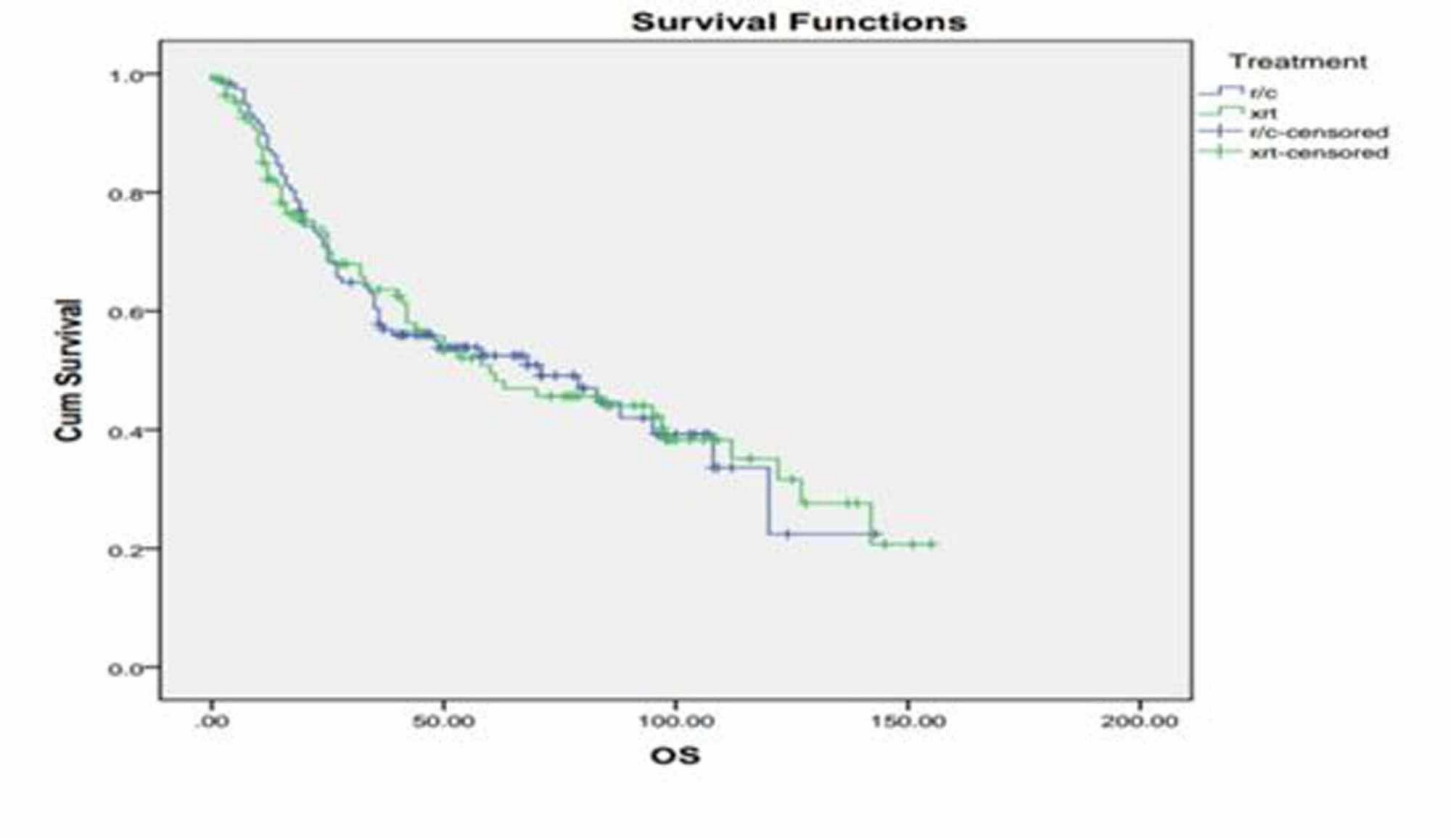
Radical radiotherapy (xrt) vs. radical cystectomy (r/c) overall survival

**Figure 3:**
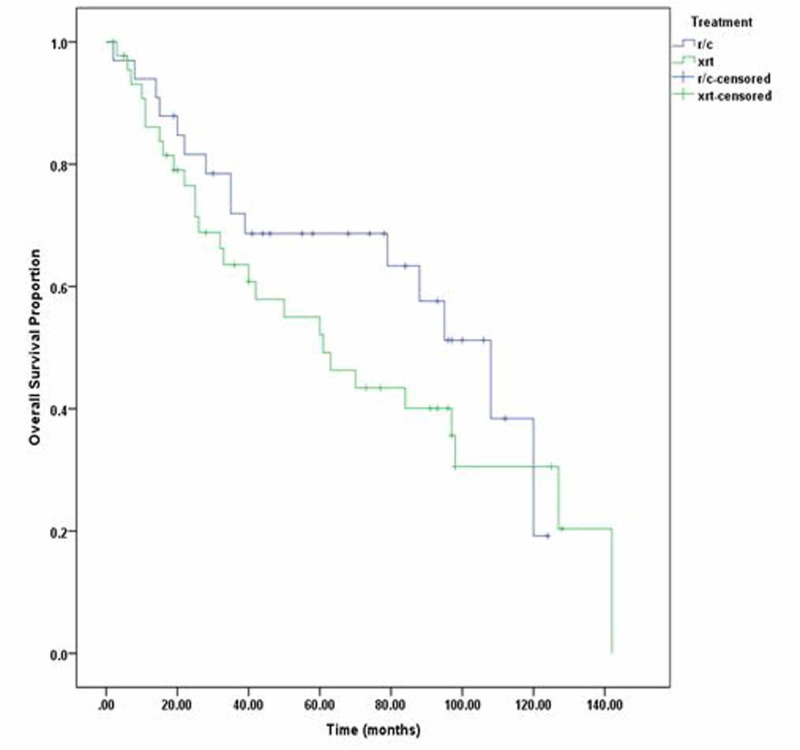
Clinical stage II radical radiotherapy (xrt) vs. radical cystectomy (r/c) overall survival

**Figure 4:**
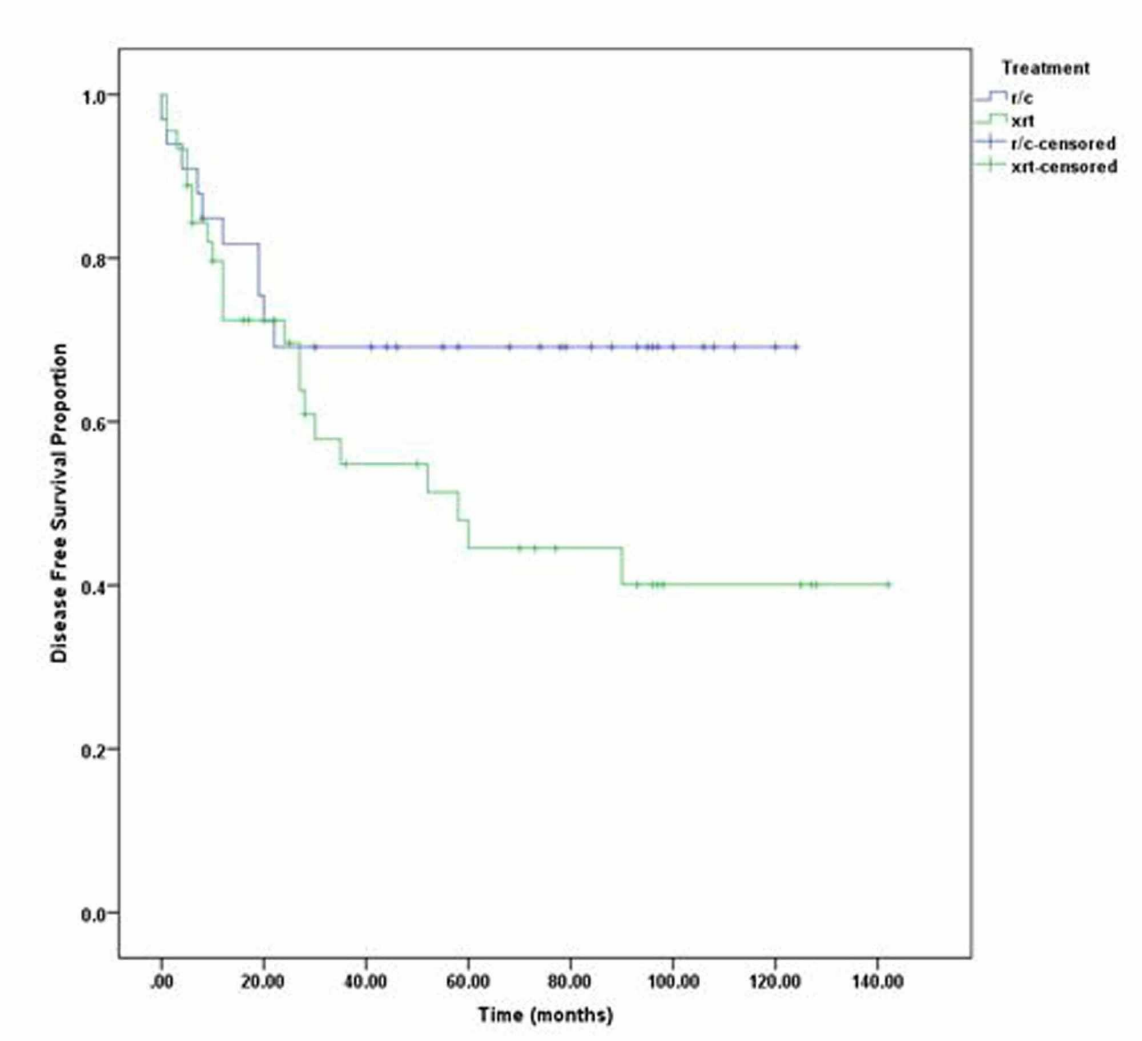
Clinical stage II radical radiotherapy (xrt) vs. radical cystectomy (r/c) disease-free survival

**Figure 5:**
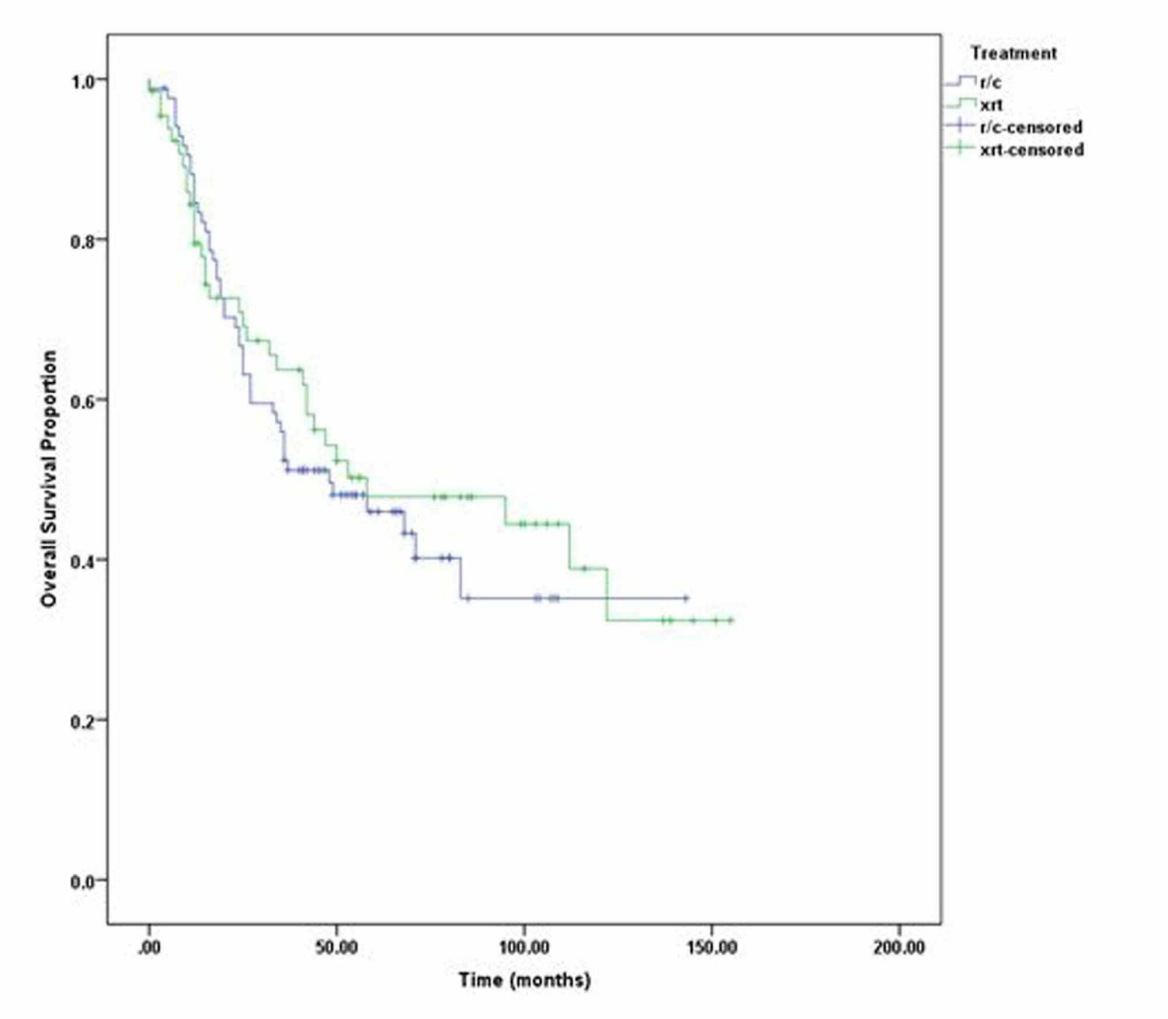
Clinical stage lII radical radiotherapy (xrt) vs. radical cystectomy (r/c) overall survival

**Figure 6:**
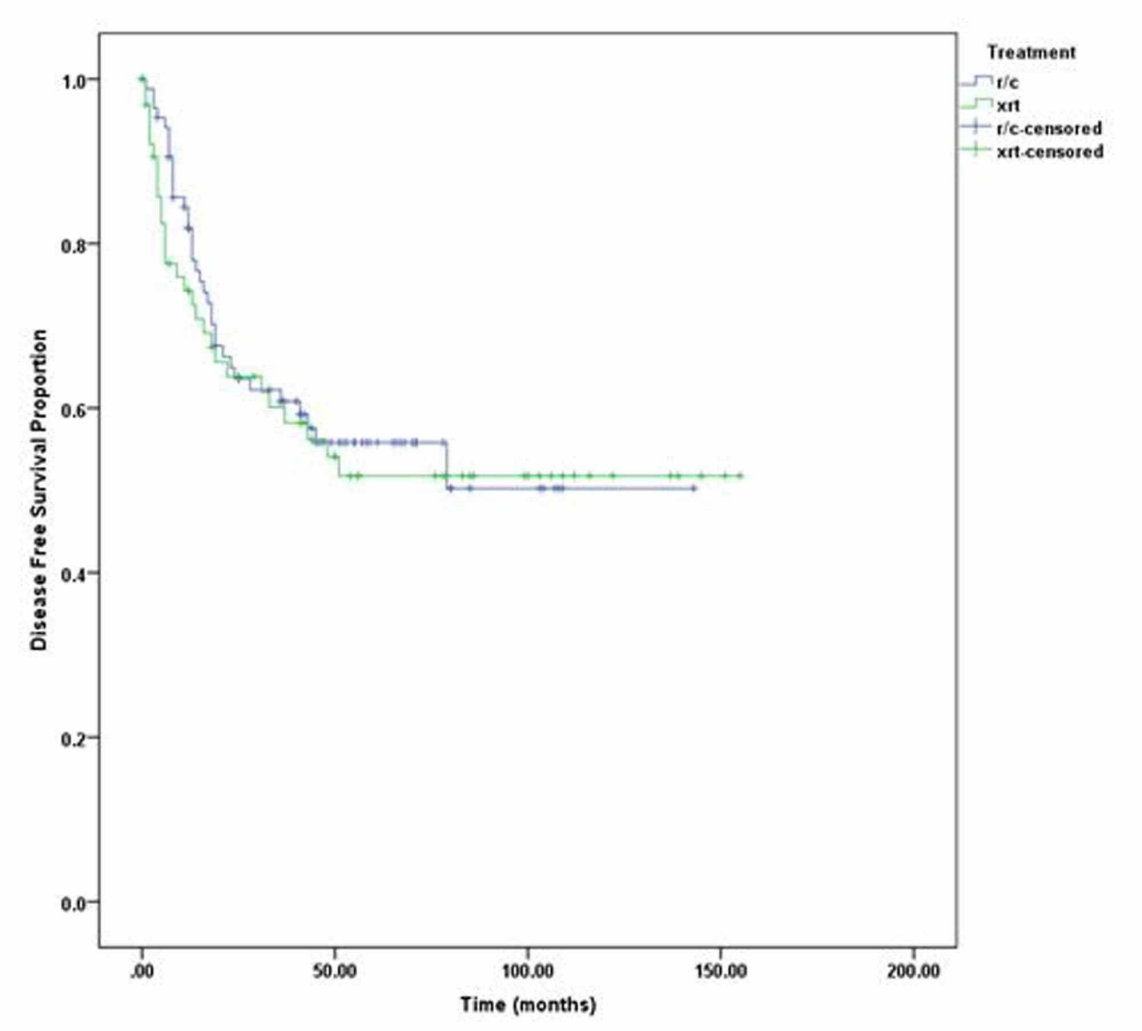
Clinical stage IlI radical radiotherapy (xrt) vs. radical cystectomy (r/c) disease-free survival

Additionally, three-year DFS of radical cystectomy was 63% and that of radiotherapy was 57%, while five-year DFS of radical cystectomy was 60% and that of radical radiotherapy was 49% (p=0.196) as shown in Figure 1. Three-year OS of radical cystectomy was 64% and radical radiotherapy was 58%; five-year OS of RC was 53% and that of radical radiotherapy was 50% (p =0.98). We also compared three- and five-year DFS and OS of stage 2 and stage 3 disease separately, but the p-value remained insignificant as shown in Figure 2. Patients with clinical stage 2 disease had OS for RC of 72% for three years and 69% for five years, while for radical radiotherapy they had 64% for three years and 55% for five years with p=0.23. Patients with clinical stage 2 disease are shown in Figure 3. DFS for RC was 69% for three years and 69% for five years, while for radical radiotherapy it was 55% for three years and 48% for five years with p=0.09 as shown in Figure 4. Patients with clinical stage 3 disease had OS for RC of 52% for three years and 46% for five years, while for radical radiotherapy they had 64% for three years and 48% for five years with p=0.56 as shown in Figure 5. Patients with clinical stage 3 disease had DFS for RC of 61% for three years and 56% for five years, while for radical radiotherapy it was 60% for three years and 52% for five years with p=0.65 as shown in Figure 6.

## Discussion

MIBC has a wide range of presentation and the most favorable treatment plan is dependent on disease stage. When making such decisions, the aim should be to achieve the most favorable oncological outcome with minimal compromise on quality of life. Clinical guidelines suggest that in patients having localized muscle-invasive bladder tumor, radical cystectomy with bilateral pelvic lymph node dissection is considered the standard treatment option. However, strategies to preserve bladder may be adopted in selected patients with intent of improved life quality. Chemo-radiotherapy is considered a suitable bladder preserving approach [10,11]. We offered primary choice of cystectomy or radiotherapy (aged patients, patients with comorbidities, or those not willing for surgery). Some of our patients initially decided on RC but later changed their decision and opted for radiotherapy. Currently the standard treatment option for MIBC is RC. The superiority of RC over radiotherapy is evident by meta-analysis of randomized trials, with five-year OS favoring surgery over radiotherapy (odds ratio 1.85, 95% confidence interval 1.22-2.82) [12].

The five-year OS of 53% for RC in our center is similar to the results of a study conducted in Taiwan with five-year OS of 53% [13] and close to results reported in Japan of 58% [14]. Another long-term study of RC has reported five- and 10-year overall survival of 58-66% and 43-44%, respectively [15].

The time delay between diagnosis and cystectomy can affect survival and delay of more than 90 days can result in worsening of survival [16]. In all our patients we performed cystectomy within two to three months. Neoadjuvant chemotherapy is considered beneficial before radical cystectomy. We noted five-year OS of 58% and 50% in patients with and without neoadjuvant chemotherapy, respectively. A study reported an overall survival advantage of 6.5% with use of neoadjuvant cisplatin-based chemotherapy [17]. Three patients of RC with ileal conduit were found to have positive urethral margins on histopathology report. They further proceeded with urethrectomy. Ten patients with pN+ (pathologically positive lymph nodes for malignancy) on histopathology were given adjuvant chemotherapy. Six patients with local recurrence were treated with palliative chemotherapy.

Growing interest in life quality has promoted the tendency towards bladder preservation strategies for MIBC. Therefore many centers have now started chemoradiation for patients who are medically fit on an elective basis in order to keep natural functional urinary bladder with improved quality of life [11,18]. In our hospital, we offered radiotherapy to patients with comorbidities and aged patients not medically fit for surgery, but radiotherapy was selected by some of the medically fit patients although they were counseled of the superior results of cystectomy. The main reason was the cystectomy-related potential complications along with the life quality concerns, regular aftercare, and external appliances like stoma bag.

In our study, 102 patients were given both chemo and radiotherapy (CRT); eight patients were given radiotherapy alone as they were considered not fit for chemotherapy. Local recurrence was noted in 16 patients. Out of these, 10 proceeded with salvage cystectomy. The remaining six had either refused surgery or were considered unfit for surgery and offered symptomatic treatment only.

Five-year OS of 50% with radiotherapy in our study is close to the Radiation Therapy Oncology Group (RTOG) study. That study analyzed long-term data of CRT and reported overall survival of 57% (five-year) and 36% (10-year) [8].

The majority of studies have found that RC is a superior option to radical radiotherapy, however there are some studies that reveal no significant difference in OS and DFS for the two options. We also compared individually both stage 2 and 3. Two-thirds of patients in our study included clinical stage 3 disease, while the remaining one-third were clinical stage 2. Oncological outcome comparison between RR and RC in each group also revealed no significant difference.

Our study findings are similar to those of Gofrit et al. [19] and Kulkarni et al. [20], who reported no significant difference between CRT and radical cystectomy in disease-specific survival and overall survival.

The data comparison between RC and radiotherapy may not be very correct if analyzed retrospectively. With such an indirect comparison a definite decision about treatment superiority cannot be made. The study can be biased due to the issue that most of the patients of the radiotherapy group had comorbid conditions or were not fit for surgery. Very few patients who were candidates for cystectomy opted for radiotherapy while the patients in the cystectomy group were medically fit. Apart from this the difference between clinical and pathological staging of radical cystectomy and radiotherapy can be different, as there is more tendency for tumor under staging in radiotherapy, which can also make the result biased.

## Conclusions

Most studies favor RC and consider it as the gold standard treatment for muscle-invasive bladder tumor treatment. The current study reveals that bladder preservation approach by chemo-radiotherapy is a viable treatment option, having comparable oncological outcomes with patients receiving radical cystectomy, and can be offered to patients having muscle-invasive urothelial bladder cancer.
